# Characterization and genetic dissection of maize ear leaf midrib acquired by 3D digital technology

**DOI:** 10.3389/fpls.2022.1063056

**Published:** 2022-12-01

**Authors:** Sheng Wu, Jinglu Wang, Yanxin Zhao, Weiliang Wen, Ying Zhang, Xianju Lu, Chuanyu Wang, Kai Liu, Bo Chen, Xinyu Guo, Chunjiang Zhao

**Affiliations:** ^1^ Information Technology Research Center, Beijing Academy of Agriculture and Forestry Sciences, Beijing, China; ^2^ Beijing Key Lab of Digital Plant, National Engineering Research Center for Information Technology in Agriculture, Beijing, China; ^3^ Beijing Key Laboratory of Maize DNA (DeoxyriboNucleic Acid) Fingerprinting and Molecular Breeding, Maize Research Center, Beijing Academy of Agriculture and Forestry Sciences, Beijing, China

**Keywords:** ear leaf, midrib spatial structure, 3D digitization, GWAS, candidate gene

## Abstract

The spatial morphological structure of plant leaves is an important index to evaluate crop ideotype. In this study, we characterized the three-dimensional (3D) data of the ear leaf midrib of maize at the grain-filling stage using the 3D digitization technology and obtained the phenotypic values of 15 traits covering four different dimensions of the ear leaf midrib, of which 13 phenotypic traits were firstly proposed for featuring plant leaf spatial structure. Cluster analysis results showed that the 13 traits could be divided into four groups, Group I, -II, -III and -IV. Group I contains HorizontalLength, OutwardGrowthMeasure, LeafAngle and DeviationTip; Group II contains DeviationAngle, MaxCurvature and CurvaturePos; Group III contains LeafLength and ProjectionArea; Group IV contains TipTop, VerticalHeight, UpwardGrowthMeasure, and CurvatureRatio. To investigate the genetic basis of the ear leaf midrib curve, 13 traits with high repeatability were subjected to genome-wide association study (GWAS) analysis. A total of 828 significantly related SNPs were identified and 1365 candidate genes were annotated. Among these, 29 candidate genes with the highest significant and multi-method validation were regarded as the key findings. In addition, pathway enrichment analysis was performed on the candidate genes of traits to explore the potential genetic mechanism of leaf midrib curve phenotype formation. These results not only contribute to further understanding of maize leaf spatial structure traits but also provide new genetic loci for maize leaf spatial structure to improve the plant type of maize varieties.

## 1 Introduction

The morphological characteristics of maize during growth and development are essential information in breeding research and field production management. The canopy structure of maize plants and the spatial distribution of leaves influence the canopy CO_2_ transport and light interception capacity, which have a great impact on growth and development, stress resistance and yield (Mantilla-Perez and Salas Fernandez, 2017; Tian et al., 2019). As the main organ for photosynthesis in maize (Strable and Nelissen, 2021), the morphology of maize leaves determines the compactness of the maize plant and directly affects the canopy interception capacity and group light energy utilization of the maize population. As the backbone of the leaf, the leaf midribs not only transport water, inorganic and organic matter and other nutrients for the maize leaf, but also directly determine the spatial orientation of the leaf (Brodribb et al., 2010; Mantilla-Perez and Salas Fernandez, 2017). In addition, the leaves will wilt in the absence of water, but the leaf midribs continue to support the leaves and maintain normal physiological functions for the leaves, thus preventing further damage to the plant caused by stress. Therefore, efficient and accurate acquisition and reconstruction of the morphological characteristics of maize leaf midrib spatial structure is of great importance for the selection and cultivation studies of dense and high-yielding varieties.

In recent years, the accuracy and acquisition range of plant phenotypic sensor technology has expanded with the development of such technology, especially with the continuous improvement of 3D digital devices. As a result, researchers can use these sensors to obtain highly accurate three-dimensional morphological structure phenotype data of plants. According to different measurement principles, three-dimensional digital equipment is mainly divided into three categories: the three-dimensional digitizer (Wang et al., 2018; Wen et al., 2018), the three-dimensional laser scanner (Garrido et al., 2015; Wu et al., 2019) and the computer vision-based system (Elnashef et al., 2019; Wu et al., 2020a). The digitizer equipment can accurately collect the key point data of plant structure, and does not rely on the complex data processing algorithm in the later stage, so it is widely used. Based on the data obtained by the three-dimensional digitizer equipment, the phenotypic parameters can be extracted directly without relying on complex post-processing algorithms, and its data accuracy is the highest, which is often used for accuracy comparison and verification of the other two types of technical equipment (Wang et al., 2018). In this study, we use a three-dimensional digitizer to obtain the ear leaf midrib spatial structure, so as to provide high-precision data guarantee for genetic analysis.

Crop phenotype is the result of complex interactions between genetic and environmental factors. A great deal of genetic diversity within species has been revealed by genome sequencing technology. However, due to the lack of phenotypic information related to genetic variation, such abundant genetic information is rarely translated into actual crop yield. Nowadays, more and more researchers are starting to perform genetic analyses based on crop phenomics. For example, genomic-assisted breeding (GAB) for crop improvement by conducting QTL mapping, association mapping and genome-wide association studies (GWAS), using phenomics data to identify genes/QTL. Among these, GWAS provides us an effective approach to explore the genetic mechanisms of phenotype formation between individuals (Xiao et al., 2016; Liu and Yan, 2018). Since the release of the maize B73 reference genome, GWAS has been widely used in maize genetics research such as traditional agronomic traits such as maize plant height (PH) and ear height (EH) (Li et al., 2016), kernel length (Dai et al., 2018), and moisture content (Zhou et al., 2018), typical traits such as microscopic phenotypes (Mazaheri et al., 2019; Zhang et al., 2021), nutrient composition (Li et al., 2013; Xu et al., 2017; Baseggio et al., 2019), metabolism (Chen et al., 2016), physiological and biochemical properties (Liu et al., 2016; Xu et al., 2018), and heavy metal enrichment (Zhao et al., 2017), and stress resistance (Zhang et al., 2016; Shi et al., 2018; Cooper et al., 2019; Wang et al., 2019; Xie et al., 2019). GWAS has contributed significantly to the elucidation of the genetic mechanism of phenotype formation in maize. However, there are few genetic studies on the phenotypes associated with leaf nerve curves in maize ear position.

In this study, 498 maize inbred lines were used to characterize the leaf midrib of maize ear position leaves. An optimal smoothing method based on curvature constraints was proposed and a multidimensional phenotype calculation method was developed to quantify 15 leaf midrib phenotypic traits covering four dimensions from 0D to 3D. To further elucidate the phenotype formation mechanism of the leaf midrib, GWAS analysis was conducted on 13 key traits. Pathway enrichment analysis of candidate genes for the traits was performed to reveal the genetic mechanism of leaf midrib curve phenotype formation.

## 2 Materials and methods

### 2.1 Materials

The materials are 498 maize inbred lines, which came from maize association analysis population (Yang et al., 2011), including 126 temperate non-stiff stalk (NSS) lines, 30 stiff stalk synthetic (SS) lines, 225 tropical/subtropical (TST) lines and 117 mixed materials. The materials were planted in the Nanfan breeding station of Maize Research Center, Beijing Academy of Agriculture and Forestry (Yazhou District, Sanya City, Hainan Province, longitude: 109.1832, latitude: 18.3623). The materials were sown on March 17, 2021, according to the unified density, the row spacing is 60cm and the plant spacing is 30 cm. Each material is planted in 2 rows, with 9 plants in each row, and is separated by 2 rows to facilitate manual sampling. The soil of the plow layer is loamy sandy soil, and the materials were not under water and nutrient stress during the growth period.

### 2.2 Data acquisition

Maize plant stops vegetative growth and enters reproductive growth in the flowering and milking stage which is considered to be the stable period of plant type structure. In practice, it is easier to determine the period by observing the silking time of maize, the maize plant reached the flowering and milking stage ~12 days after silking. And the maize varieties with stable plant types were selected in turn for data acquisition. From May 18—27, 2021, we took samples from the field and used two digitizer equipment (Polhemus FASTSCAN, USA) to obtain data synchronously, A total of 1579 samples were obtained, with no less than 3 repetitions for each material. To obtain high-quality leaf midrib phenotype data, the field sampling and data acquisition process is as follows: (1) Field sampling, the experimenter dug up the samples with their roots and transported them to the level road near the field; (2) Transplant sample, the samples were transplanted into carrier pots with an appropriate amount of water; (3) Transport samples, the samples were transported to the data collection room by tricycle, and the plant was held by experimenter to avoid sample damage during transportation; (4) Manual repair, for the broken leaf midrib, use a fixing clip to fix at the broken place, and for the broken leaf base, use a support nail to fix on the outside of the leaf base. From sampling to the end of data collection, the time for each sample is controlled within 30 minutes.

**Figure 1:**
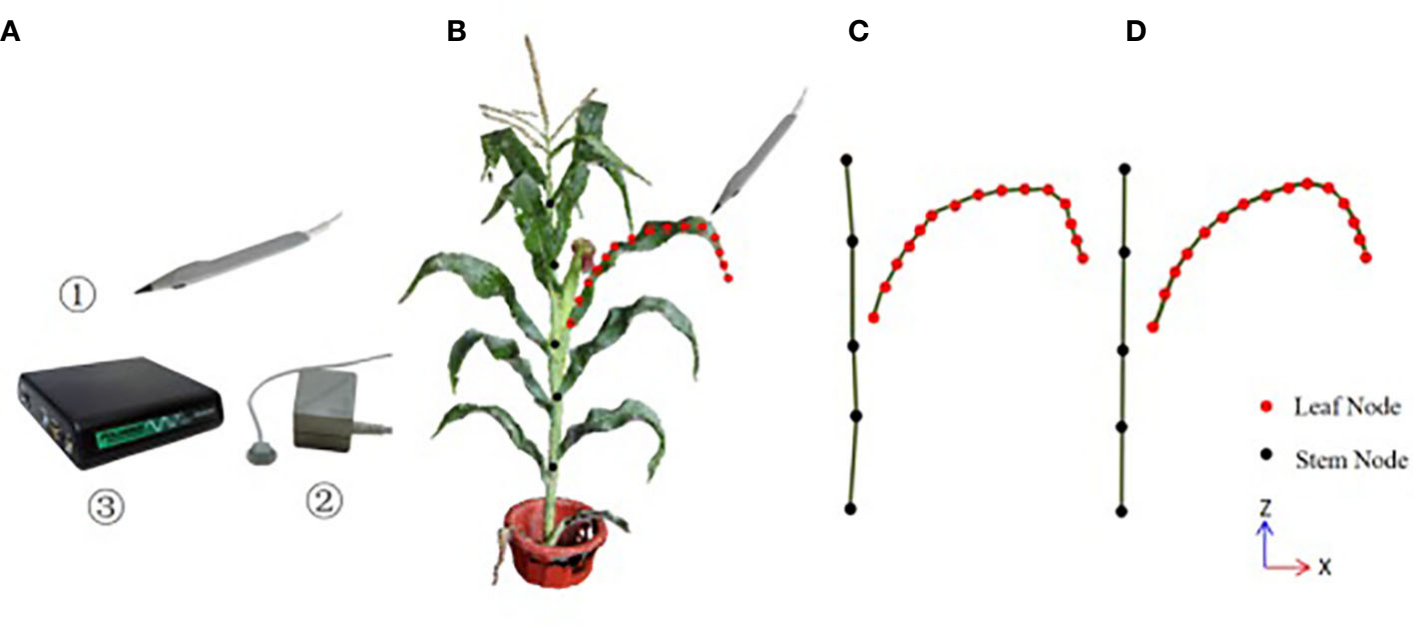
The three-dimensional digitizer device and the schematic diagram of three-dimensional digital key point acquisition of corn ear leaf **(A)** Polhemus FASTSCAN, ①the probe pen, ②the electromagnetic emission locator, ③ the position calculation unit; **(B)** the acquisition rules of the digitizer, **(C)**the topological relationship of stalk node key points and leaf midrib key points collected by the digitizer, **(D)** the results of the key points of stalk nodes and leaf midribs after smooth correction by the method in this paper.

The three-dimensional digitizer device Polhemus FASTSCAN is used for data acquisition in this paper, which consists of three parts: a probe pen, an electromagnetic emission locator and a position calculation unit, whose accuracy is 0.25cm, as shown in **Figure 1A**. The experimenter selects the key point position data through the probe pen. The acquisition rules of 3D digital key points are shown in **Figure 1B**. Firstly, the key points of stalk nodes are collected, and *M* key nodes of stalk nodes (*S*_*m*_ , usually *M = 5*) are collected from bottom to top along the stalk nodes near the ear leaves and saved as stalk node files; Then, the key points of leaf midribs are collected (if there are multiple ears, take the attached leaves on the uppermost ears), and *N* key points of leaf midribs(*L*_*n*_ ) are collected from the leaf base, along the leaf midribs to the leaf tip, and saved as leaf midrib files, and the N value is based on the three morphological characteristics of leaf length, curvature and twist, and more key points are collected in the position with large curvature and distortion, to retain the spatial attitude details of leaf midribs. Orderly connect the key points of the stalk node and leaf midrib, as shown in **Figure 1B**, in which the orange point is the stalk point and the red point is the leaf midrib point.

150 samples were randomly selected, the leaf length, leaf angle and leaf vertical height were measured manually to verify the accuracy of phenotypic traits. First, the leaf angle at the base of the leaf was measured with an angle ruler, then the horizontal board was used to mark the horizontal projection point of the highest point of the leaf midrib on the stalk, and the vertical height was measured from the projection point to the base of the leaf. Finally, cut off the leaf and flatten it, and measure the leaf length along the vein.

### 2.3 Leaf midrib curve smoothing and correction

**Figure 2:**
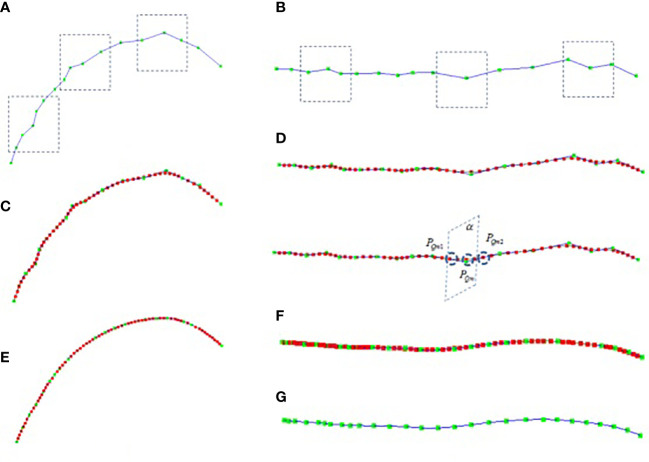
Leaf midrib curve smoothing, **(A)** the front view of collected midrib curve key points, **(B)** top view of collected midrib curve key points, **(C)** the front view of midrib curve smoothed based on cubic B-spline, **(D)** the top view of midrib curve smoothed based on cubic B-spline, **(E)** the front view of midrib curve smoothed after 8 iterations, **(F)** the top view of midrib curve smoothed after 8 iterations, **(G)** the top view of leaf midrib curve after 8 iterations of smoothing (excluding interpolation nodes).

There are two kinds of errors in the key node data obtained by using the digitizer, which are often caused by manual operation errors and the calibration problem of the digitizer. One kind of error is the positive Z-axis error of the stalk, as shown in **Figure 1B**, the collected stalk nodes are offset in the z-axis direction (If the key points are collected on different sides of the stalk node, the node deviates from the midpoint of the stalk node. Another error is that the key points of the leaf midrib curve have local fluctuation deviations (see **Figure 2A** in the vertical direction and **Figure 2B** in the horizontal direction), and this deviation is due to the unstable use of the digitizer probe by the experimenter. The deviation of key point data will reduce the accuracy of leaf midrib phenotypic parameters, so a batch algorithm pipeline is constructed to automatically correct these two kinds of problems.

#### 2.3.1 Positive direction correction

In the natural state, the maize stalk is upright, take advantage of this morphological feature. the spatial fitting line of the stalk node is calculated by the least square method, in which the stalk node sequence is expressed as *S*_*m*_(*P*_0_,*P*_1_,…,*P*_*m*_) and the spatial fitted line is expressed as *L*_*stem*_ . Further, the rotation matrix *M_stalk_* of the spatial fitted line to the Z-axis mapping is calculated, the key points of the stalk nodes are corrected to the Z-axis direction by *S_m_* *M_stalk_, at the same time, the key points of the leaf midribs are also corrected, and the results are shown in **Figure 1C**.

#### 2.3.2 Leaf midrib curve smoothing

The midrib leaf is the main supporting structure of the leaf, which controls the leaf shape and growth direction. The midrib curve presents smooth characteristics in the natural state. To eliminate the fluctuation error of key points of the midrib curve, the cubic B-spline curve smoothing algorithm is constructed to locally smooth the midrib curve. Then, the cubic B-spline iterative smoothing algorithm based is proposed to correct key points of the midrib leaf.

##### 2.3.2.1 Local smoothing of leaf midrib curve based on cubic B-spline

The commonly used quadratic curve or cubic curve is difficult to accurately characterize the corn leaf midrib curve. The B-spline model is widely used to smooth and construct complex smooth curves (Wang et al., 2013; Li et al., 2018). It is composed of a series of key point controls and has a good local smoothing optimization effect. The general construction equation of the B-spline model is shown in Formula 1:


(1) $$P(t)=\sum_{i=0}^{n}P_{i}F_{i,k}(t)$$

Where *P*_*i*_ is the control point of the curve and *F*_*i*,*k*_(*t*) is the B-spline basis function of order K. The basis function of cubic B-spline is shown in formula 2.


(2) $$F_{i,k}(t)=\frac{1}{k!}{\sum_{m=0}^{k-i}\left(-1\right)}^{m}(\begin{array}{c} m\\ k+1 \end{array}){\left(t+k-m-j\right)}^{k}$$

Where, *j* is the index value of the control point, *k* is the order, and *k*=3 . The calculation formula of each component after the expansion of the basis function is as follows:


(3) $$\begin{array}{l} F(t)=P_{0}*F_{0,3}(t)+P_{1}*F_{1,3}(t)+P_{2}*F_{2,3}(t)+P_{3}*F_{3,3}(t)\\ F_{0,3}(t)=\frac{1}{6}{\left(1-t\right)}^{3}\\ F_{1,3}(t)=\frac{1}{6}(3t^{3}-6t^{2}+4)\\ F_{2,3}(t)=\frac{1}{6}(-3t^{3}+3t^{2}+3t+1)\\ F_{3,3}(t)=\frac{1}{6}t^{3} \end{array}$$

The key points of the leaf midrib curve collected based on the three-dimensional digitizer are expressed as *L*(*P*_0_,*P*_1_,…,*P*_*n*_) , as shown in **Figure 2A**, the green node is the key point. The local smoothing algorithm flow of leaf midrib curve based on cubic B-spline is as follows: Firstly, the key points are interpolated equidistantly to make the distance between the key points equal, for each segment *P*_*m*_ *P*_*m*+1_ , calculate the segment length *D*_*m*_ , and the number of nodes inserted between *P*_*m*_ *P*_*m*+1_ is *N*=*ceil*(*D*_*m*_/*D*_*b*_)−1 , where *ceil* is the rounding operator, *D*_*b*_ is the segment length after interpolation(*D*_*b*_=4*cm* ). After equidistant interpolation, the curve key points are expressed as *L*1(*P*_0_,*P*_1_,…,*P*_*h*_) . Then, for each segment *P*_*m*_ *P*_*m*+1_ , smooth interpolation is carried out based on cubic B-spline, and the number of interpolation nodes is *c* (default parameter, c=30 ), that is *P*_*m*_ *P*_*m*1_…*P*_*mg*_…*P*_*mc*_ *P*_*m*+1_ , for each interpolation point *P*_*mg*_ , the interpolation points of the smoothed midrib curve are calculated in sequence through formula 3, where, *t*=*g*/(*c*+1) . The smoothed midrib curve is *L*2(*P*_0_,*P*_1_,…,*P*_*h*+(*h*−1)*c*_) as shown in **Figure 2C, D**-1, and the red node in the figure is the smoothed node. It can be seen that the local smoothing effect of the leaf midrib curve has been improved after cubic B-spline smoothing.

##### 2.3.2.2 Global smoothing of leaf midrib curve based on curvature

Based on the cubic B-spline curve smoothing algorithm, the local smoothing effect of the leaf midrib curve is improved, but the smoothed curve still has the fluctuation error in horizontal plane and vertical plane. The curve curvature represents the degree of curve bending, and the curvature value of the key point of the curve is the rotation speed of the tangent vector at the point to the arc length. At the fluctuation node of the curve, the curvature value is usually large, so the cubic B-spline iterative smoothing algorithm of the midrib curve based on curvature constraint (CISCB-spline) is constructed for the global smoothing of the midrib curve. The general formula for curve curvature calculation is as follows:


(4) $$\kappa (t)=\frac{\left|{\vec{r}}^{\prime }(t)\times {\vec{r}}^{\prime \prime }(t)\right|}{{\left|{\vec{r}}^{\prime }(t)\right|}^{3}}$$

For 3D space (*C* ^3^ ) curve, and its parameter equation as:


(5) $$\vec{r}(t)={\left(x(t),y(t),z(t)\right)}^{T}$$

Based on Formula 4 and 5, the calculation formula of leaf midrib curve curvature is as follows:


(6) $$\kappa (t)=\frac{\sqrt{(z^{\prime \prime }(t)y^{\prime }(t)-{\left(y^{\prime \prime }(t)z^{\prime }(t)\right)}^{2}+(x^{\prime \prime }(t)z^{\prime }(t)-{\left(z^{\prime \prime }(t)x^{\prime }(t)\right)}^{2}+(y^{\prime \prime }(t)x^{\prime }(t)-{\left(x^{\prime \prime }(t)y^{\prime }(t)\right)}^{2}}}{{\left({x^{\prime }}^{2}(t)+{y^{\prime }}^{2}(t)+{z^{\prime }}^{2}(t)\right)}^{3/2}}$$

Based on cubic B-spline formula 3, the partial calculation formula of X, Y and Z is as follows:


(7) $$\begin{array}{l} x(t)=F_{x}(t)=P_{0,x}*F_{0,3}(t)+P_{1,x}*F_{1,3}(t)+P_{2,x}*F_{2,3}(t)+P_{3,x}*F_{3,3}(t)\\ y(t)=F_{y}(t)=P_{0,y}*F_{0,3}(t)+P_{1,y}*F_{1,3}(t)+P_{2,y}*F_{2,3}(t)+P_{3,y}*F_{3,3}(t)\\ z(t)=F_{z}(t)=P_{0,z}*F_{0,3}(t)+P_{1,z}*F_{1,3}(t)+P_{2,z}*F_{2,3}(t)+P_{3,z}*F_{3,3}(t) \end{array}$$

The cubic B-spline iterative smoothing algorithm flow of leaf midrib curve based on curvature constraint is described in detail. Firstly, based on the curve curvature calculation formula 6, the curvature corresponding to each node of the curve *L*2(*P*_0_,*P*_1_,…,*P*_*h*+(*h*−1)*c*_) after cubic B-spline smooth interpolation is calculated; And the interpolation points with curvature greater than the threshold *κ*_*thre*_ are found, where *κ*_*thre*_ is a user-defined parameter (default parameter, *κ*_*thre*_=0.004 ), and, the node sequence {*P*_*Q*1_,*P*_*Q*2_,…*P*_*Qm*_,…*P*_*Qn*_} in the corresponding key points *L*1(*P*_0_,*P*_1_,…,*P*_*h*_) of leaf midrib curve is constructed according to the number C of cubic B-spline smooth interpolation points; Then, the node *P*_*Qm*_ are sequentially modified according to the following method. The correction plane *α* is constructed with two nodes *P*_*Qm*1_ and *P*_*Qm*2_ , which are the front and rear connection nodes of *P*_*Qm*_ , as the plane normal vector (as shown in **Figure 2D**-2). The intersection $P_{Qm}^{\prime }$ of the curve sequence segment *L*2(*P*_0_,*P*_1_,…,*P*_*h*+(*h*−1)*c*_) and the plane *α* is calculated. The modified node sequence $\left\{P_{Q1}^{\prime },P_{Q2}^{\prime },\ldots P_{Qm}^{\prime },\ldots P_{Qn}^{\prime }\right\}$ corresponding to the node sequence {*P*_*Q*1_,*P*_*Q*2_,…*P*_*Qm*_,…*P*_*Qn*_} is calculated by this method. Finally, the node position corresponding to the key points *L*1(*P*_0_,*P*_1_,…,*P*_*h*_) of the leaf midrib curve is updated. The above steps are iterated until the corresponding curvature of all smoothed nodes is less than the threshold *κ*_*thre*_ or the number of iterations is greater than h times (h = 20 is set in this paper). The smoothing results are shown in **Figure 2E–G**, and after 8 iterations, it has a good smoothing effect in the global range, and the deviations of key nodes in the horizontal and vertical directions are corrected.

### 2.4 Leaf midrib phenotype

The different morphological structures of maize leaf midribs are affected by factors such as planting environment, space competition and maize variety characteristics. In order to quantitatively calculate the spatial phenotypic indexes of leaf midribs, before calculating the phenotypic parameters, the growth direction correction and leaf midrib direction plane normalization should be carried out for the key points of leaf midribs. The growth direction correction is to ensure that the growth direction of corn plants is in the z-axis direction, and the leaf midrib direction plane normalization is to ensure that the leaf midrib direction plane is in the *XOZ* plane direction. The growth direction correction algorithm has been described in section 2.3.1.

#### 2.4.1 Leaf midrib direction plane normalization

**Figure 3:**
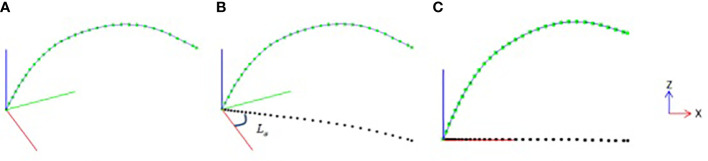
Leaf midrib direction plane normalization, **(A)** leaf midrib curve after smooth correction, **(B)** projection point of leaf midrib curve on *XOY* plane, **(C)** leaf midrib curve after normalization of direction plane.

In order to quantitatively calculate the growth characteristics of the leaf midrib curve in three-dimensional space, the smoothed leaf midrib curve is corrected by normalized direction. First, the nodes of the leaf midrib curve are projected on the *XOY* plane (**Figure 3B**), and the projection point set is defined as *L*_*pro*_ . Then, the straight line *L*_*p*_ is fitted from the point set *L*_*pro*_ based on the least squares algorithm. Through the angle between the straight line *L*_*p*_ and *OX-*axis, the rotation matrix *M*_*xoz*_ from the midrib direction plane to the *XOZ* plane is calculated, and the positive direction of the midrib curve is normalized based on the rotation matrix *M*_*xoz*_ (**Figure 3C**).

#### 2.4.2 Extract leaf midrib phenotypic parameters

**Figure 4:**
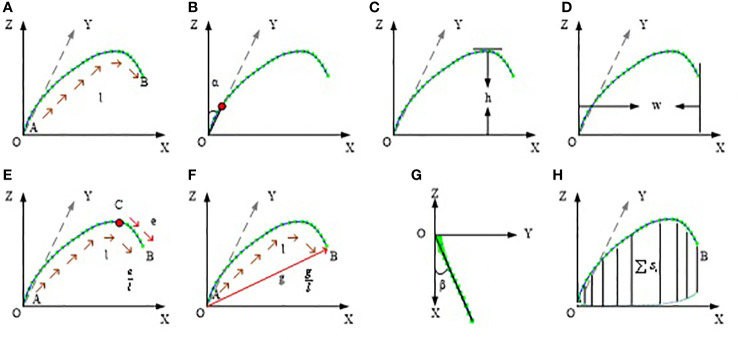
Schematic diagram of leaf midrib phenotypic traits. **(A)** leaf length, **(B)** leaf angle, **(C)** leaf upward growth height, **(D)** leaf horizontal growth length, **(E)** upper leaf midrib sag, **(F)** leaf midrib curvature, **(G)** leaf midrib deviation, **(H)** leaf midrib space occupied area.

Based on the corrected leaf midrib curve, the multi-dimensional leaf midrib phenotype indexes are calculated in three-dimensional space. In the four dimension categories, a total of 15 phenotypic traits are obtained, and the calculation method is shown in **Table S1**. Among them, 0-d index is a bool value, which indicates whether the leaf tip is drooping; 1-D index is leaf length(**Figure 4A**), which has a greater correlation with leaf biomass; 2-D index includes leaf angle(**Figure 4B**), leaf upward growth height(*VerticalHeight*, **Figure 4C**), and leaf horizontal growth length(*HorizontalLength*, **Figure 4D**), which are used to quantify leaf compactness; and 3-D index includes 8 phenotypic parameters: upper leaf midrib sag(*DeviationTip*, **Figure 4E**), leaf midrib curvature(*CurvatureRatio*, **Figure 4F**), maximum curvature value, maximum curvature position ratio, leaf midrib deviation(*DeviationAngle*, **Figure 4G**), maximum disturbance rate, maximum winding rate position ratio, and leaf midrib space occupied area(*ProjectionArea*, **Figure 4H**), which are used to quantify leaves curvature, deflection and space occupation.

#### 2.4.3 Leaf midrib phenotype system

Leaf midrib phenotype system (LMPS) is developed based on the algorithm in this paper, using the C++ language, and which runs on Windows 10 operating system, processor Intel Core i7, CPU 3.6GHz, memory 8GB. The algorithm flow of LMPS is shown in **Algorithm file S2**.

### 2.5 Statistical analysis of leaf midrib phenotypic data

Descriptive statistical analysis of phenotypic data was performed by R (Version 3.6.3) software (https://cran.r-project.org/). To determine whether the phenotypes differed between subpopulations, an analysis of variance (ANOVA) was performed in this study. To explore the relationships between phenotypes, Pearson correlation analysis was used to calculate correlation coefficients for phenotypic traits. *Pheatmap*, a function of R package “pheatmap”, was applied to perform clustering based on Pearson correlation coefficient as a distance measure, and a heatmap was used to show the strength of the correlation between the traits. Repeatability of traits was calculated by the *asreml* function of R package “asreml” (ASReml-R v.4.0) (Butler, 2009). The traits with high repeatability were used for GWAS.

### 2.6 Genome-wide association analysis

The genotypic data used in this study were obtained from Maizego (download URL: www.maizego.org/Resources.html). For the genotypic data of 498 lines, SNP markers with minimum allele frequency (MAF) less than 0.05 and call rate less than 0.9 were removed by PLINK 1.09 software. Then 778,640 SNPs passed quality control and were used for GWAS. The GWAS in this study was conducted by a multi-locus random-SNP-effect mixed linear model tool “mrMLM” (version 4.0) (Zhang et al., 2019) that included six GWAS methods (mrMLM, FASTmrMLM, FASTmrEMMA, ISIS EM-BLASSO, pLARmEB and pKWmEB). To control the false positives of GWAS, population structure estimated by Admixture (Alexander et al., 2009) and relative kinship calculated by TASSEL 5 (Bradbury et al., 2007) were added to the model. Finally, the results obtained by all six GWAS methods with *P*-value<0.5/N (where N is the total number of genome-wide SNPs) were used as significant SNPs associated with each phenotypic trait (Zhang et al., 2021). Furthermore, the most significant SNP obtained for each method was indicated by Top 1, and the overlapped SNPs of multiple GWAS methods were used to be significant results with high reliability. Candidate genes were identified by ANNOVAR (Wang et al., 2010) with the main function “annotate_variation.pl”, and the maize reference database was constructed based on the maize B73 reference genome (B73 RefGen_v4) available in EnsemblPlants (http://plants.ensembl.org/Zea_mays/Info/Traits). With default parameters, a gene if a significant SNP overlaps its coding (exonic) or intron (intronic), or overlaps its 5’ untranslated region (UTR5) or 3’ untranslated region, or overlaps its 1-kb region upstream/downstream of transcription start/end site (upstream/downstream), or in its intergenic region (intergenic) or within 2-bp of a splicing junction (splicing), was identified as a candidate gene. Further, the NCBI Gene database (https://www.ncbi.nlm.nih.gov/gene) was used for functional annotation of candidate genes to obtain a more detailed gene description.

### 2.7 GO and KEGG pathway analysis of *candidate genes*

The biological functions of candidate genes with high confidence for each phenotypic trait (Top1 SNP annotation or multiple GWAS validation) were explored by pathway enrichment analysis. Enrichment analysis of Gene Ontology (GO) (Ashburner et al., 2000) was conducted using PlantRegMap (Kanehisa, 2002). KOBAS V3.0 (Jin et al., 2015) was used to enrich KEGG (Bu et al., 2021) pathway. The threshold for significant results is set to *P*-value< 0.05.

In order to have a better view of the relationship between each trait and its candidate genes, an open-source software platform (Cytoscape v3.7.2) (Shannon et al., 2003) was used to display a complex trait-candidate gene-pathway network.

In this study, pathway enrichment analysis was used to explore the biological functions of candidate genes. For candidate genes annotated by the SNP with the highest significance or identified by multi-methods, enrichment analysis of Gene Ontology (GO) (Ashburner et al., 2000) terms and KEGG (Kanehisa, 2002) pathways were conducted using PlantRegMap (Jin et al., 2015) and KOBAS V3.0 (Bu et al., 2021) respectively. The threshold for significant results is set to *P*-value< 0.05.

To better demonstrate the relationship between the leaf midrib curve traits and their candidate genes, an open-source software platform (Cytoscape v3.7.2) (Shannon et al., 2003) was used to display a complex trait-candidate gene-pathway network.

## 3 Result

The data of 1579 leaf midrib curve samples were processed automatically in batch by the LMPS, the efficiency is 0.1 s per sample. Nine of the samples were automatically identified as broken leaf midribs when node curvature more than 0.2, 1570 samples were valid. The average number of repetitions of varieties is 3.15 samples per variety. A total of 15 phenotypic traits were extracted from the leaf midrib curve.

### 3.1 Visualization effect and classification

**Figure 5:**
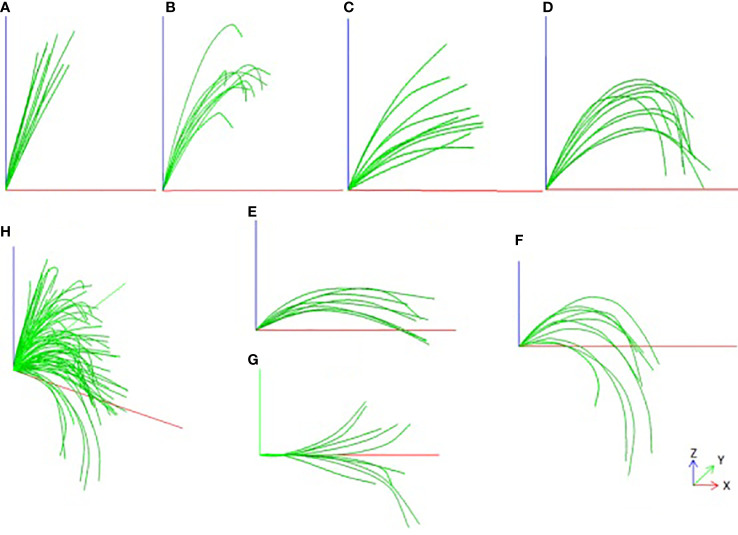
Smooth visualization effect of leaf midrib curve and Classification, **(A)** Vertical type, **(B)** Tip curved type, **(C)** Stretch type, **(D)** Bending type, **(E)** Creeping type, **(F)** Drooping type, **(G)** Deflection type, **(H)** All types.

The result of the smoothed leaf midrib curve is shown in **Figure 5**. From the results, the key points of the deviation are well corrected, not only in the local position, but also in the global leaf midrib structure, which is in line with the growth law of corn leaf midribs. It is found that the leaf midrib morphology of maize varieties shows strong diversity, there are great differences among varieties, and some varieties show similarities. According to the spatial structure, the leaf midrib curve can be divided into seven types: vertical type, tip curved type, stretch type, bending type, deflection type, creeping type and drooping type. Among them, the vertical type is the upward vertical leaf midrib with a small leaf angle; the tip curved type is the leaf midrib that bends at the tip, but stands upright from the leaf base to the highest point, depicting the characteristics of the tip; the stretch type is the leaf midrib with large leaf angle and small curvature; the bending type is the leaf midrib with large curvature; the creeping type is the leaf midrib creeping on the horizontal plane, and its vertical height is small; the drooping type is a leaf midrib with large curvature, and the leaf tip droops below the leaf base; the deflection type refers to the leaf midrib that deviates gradually from the leaf base to the side, and the direction of leaf midrib is changed. It should be noted that the deflection type leaf midrib curve may also be the other six types, and the other six types are independent of each other. The leaf midrib curves were automatically classified based on the calculated phenotypic parameters, and the classification algorithm is presented in **Algorithm file S3**.

#### 3.1.1 Subgroup distribution

**Figure 6:**
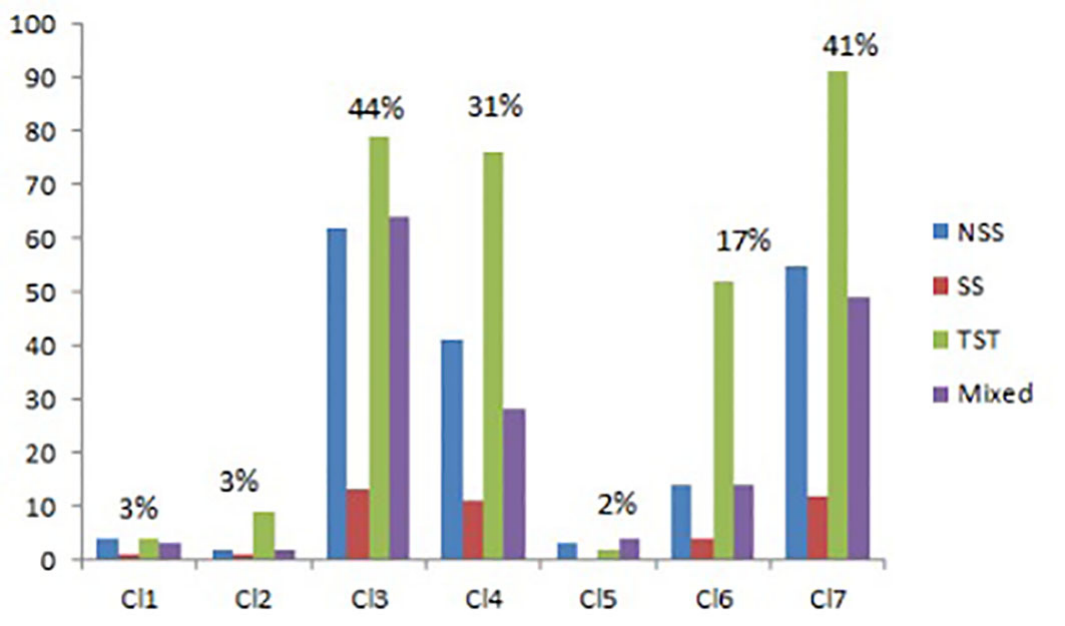
The classification and subgroup distribution of leaf midrib curve. CL1, vertical type; Cl2, Tip curved type; CL3, Stretch type; CL4, Bending type; CL5, Creeping type; CL6, Drooping type; CL7, Deflection type.

For different types of leaf midrib curves, statistical analysis is carried out according to the proportion types and the distribution subgroups. The results are shown in **Figure 6**. In terms of type proportion, there are many varieties of stretch type and bending type, accounting for 44% and 31% respectively, followed by drooping type, accounting for 17%, and the proportion of the other three types is low and close. The deflection leaf midrib curve accounted for 41% of all varieties (*DeviationAngle* > 10°). In terms of subgroup distribution, for all classifications, the number of varieties of the TST subgroup is the largest, followed by the NSS subgroup, mixed subgroup and the SS subgroup, respectively. For the stretch type classification, the number of varieties of the TST subgroup is similar to the NSS and mixed subgroup, but in other types, the number of varieties of the TST subgroup is much more than other subgroups.

#### 3.1.2 Accuracy evaluation

**Figure 7:**
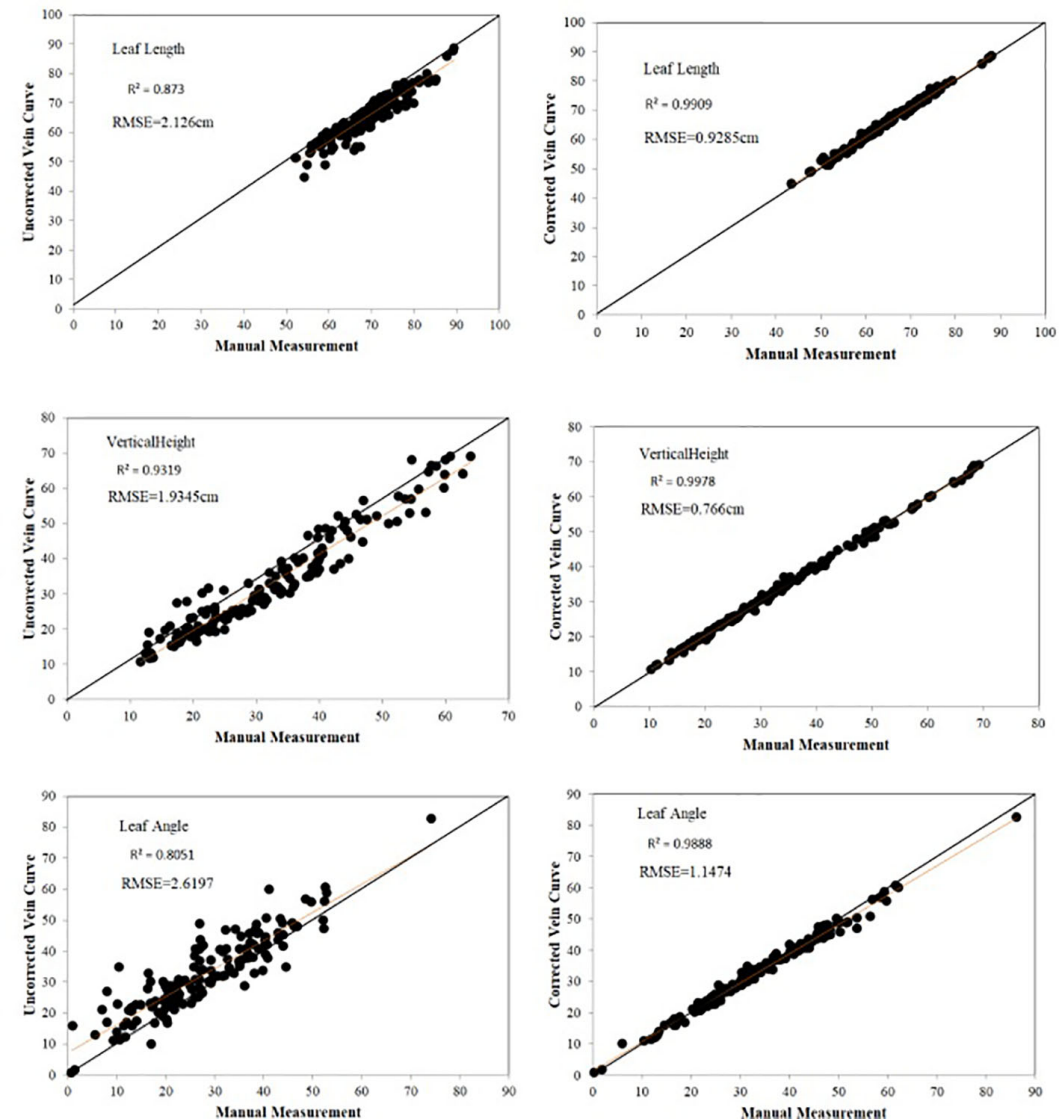
Evaluation of leaf midrib phenotypic error.

In order to improve the efficiency of data acquisition, the leaf midrib curve is manually collected at certain distance intervals using digitizer equipment, so, leaf midrib curves are not completely digitized, but only some key nodes. In addition, due to the manual operation of the probe, some key points deviate from the leaf midrib curve. Therefore, there is a certain error in calculating the leaf midrib phenotypic parameters directly from the collected leaf midrib key point data, so it is necessary to smooth and correct the leaf midrib curve (**Figure 2**).150 leaf midrib samples are selected for manual measurement, and the three phenotypic parameters of leaf length, leaf vertical height and leaf angle are measured to evaluate the necessity of leaf midrib curve smoothing and the error accuracy of this method. The accuracy comparison results are shown in **Figure 7**. Compared with manual measurement error, using unsmoothed data, the *R^2^* values of leaf length and leaf angle are less than 0.9, which are 0.873 and 0.805 respectively, while using smoothed data, the *R^2^* values of leaf length, leaf vertical height and leaf angle are more than 0.98, which are 0.991, 0.997 and 0.988. In addition, before smooth correction, the *RMSE* of leaf length, leaf vertical height and leaf angle are 2.126cm, 1.934cm and 2.619°, while after correction, they are 0.928cm, 0.766cm and 1.147°.

Data analysis showed that there are some errors using uncorrected digital data. From the visualization results of the leaf midrib curve, the key points at the base of the leaf midrib and the key points at the upper part are easy to produce errors. Among them, the key points at the base have a great impact on leaf angle, and the key points at the upper part of leaf midrib and leaf tip have a great impact on leaf length and other indexes. The data analysis shows that the phenotypic index error is greatly improved by using this method. From the visualization results of the leaf midrib curve (**Figure 2** and **Algorithm file S3**), the key points at the base and upper part of the leaf midrib are easy to deviate from the leaf midrib. Among them, the key points at the base have a great influence on the leaf angle. The key points at the upper part of the leaf midrib and leaf tip have a great influence on leaf length and other indexes.

### 3.2 Analysis of phenotypic data

Descriptive statistical analysis and normal distribution test showed that the phenotypic data of ear leaf midrib curves had a wide range of phenotypic variation in all traits (**Table 1**). The variation of traits with various dimensions was also different. TipTop, a dichotomous variable, which had no dimension (0D), characterizes whether the leaf was pendulous or not, i.e., the degree of leaf stiffness, with a coefficient of variation of 1.60. LeafLength, a 1D trait, was a typical quantitative trait, with a coefficient of variation of 0.13. The five 2D traits were also normally distributed quantitative traits, with coefficients of variation ranging from 0.20 to 0.42. The eight 3D traits were more variable, with coefficients of variation ranging from 0.15 to 13.65. Among these, MaxWindingRate had the largest coefficient of variation (13.65), indicating that this trait might be more variable and its formation might be more susceptible to environmental influences.

**Table 1:** Descriptive statistical analysis results of 15 maize leaf midrib curve traits.

Trait dimension	Trait name	Mean	Min	Max	Range	Var	SD	CV
0D	TipTop	0.28	0.00	1.00	1.00	0.20	0.45	1.60
1D	LeafLength	64.79	39.44	95.09	55.65	74.25	8.62	0.13
2D	LeafAngle	38.65	0.26	152.34	152.09	259.27	16.10	0.42
2D	HorizontalLength	45.48	2.65	84.25	81.61	117.98	10.86	0.24
2D	OutwardGrowthMeasure	0.70	0.04	0.97	0.93	0.02	0.14	0.20
2D	UpwardGrowthMeasure	0.47	0.09	0.98	0.89	0.03	0.18	0.38
2D	VerticalHeight	30.32	5.44	82.51	77.06	154.37	12.42	0.41
3D	CurvaturePos	0.52	0.17	0.83	0.67	0.03	0.17	0.32
3D	CurvatureRatio	0.84	0.34	1.00	0.66	0.02	0.13	0.15
3D	DeviationAngle	7.67	0.00	86.39	86.39	58.97	7.68	1.00
3D	DeviationTip	0.30	0.00	1.00	1.00	0.06	0.25	0.82
3D	MaxCurvature	0.08	0.00	1.37	1.37	0.01	0.10	1.27
3D	MaxWindingRate	0.07	0.00	35.93	35.93	0.95	0.97	13.65
3D	ProjectionArea	824.10	84.20	2563.10	2478.86	162732.81	403.40	0.49
3D	WindingRatePos	0.46	0.17	0.92	0.75	0.05	0.22	0.47

**Figure 8:**
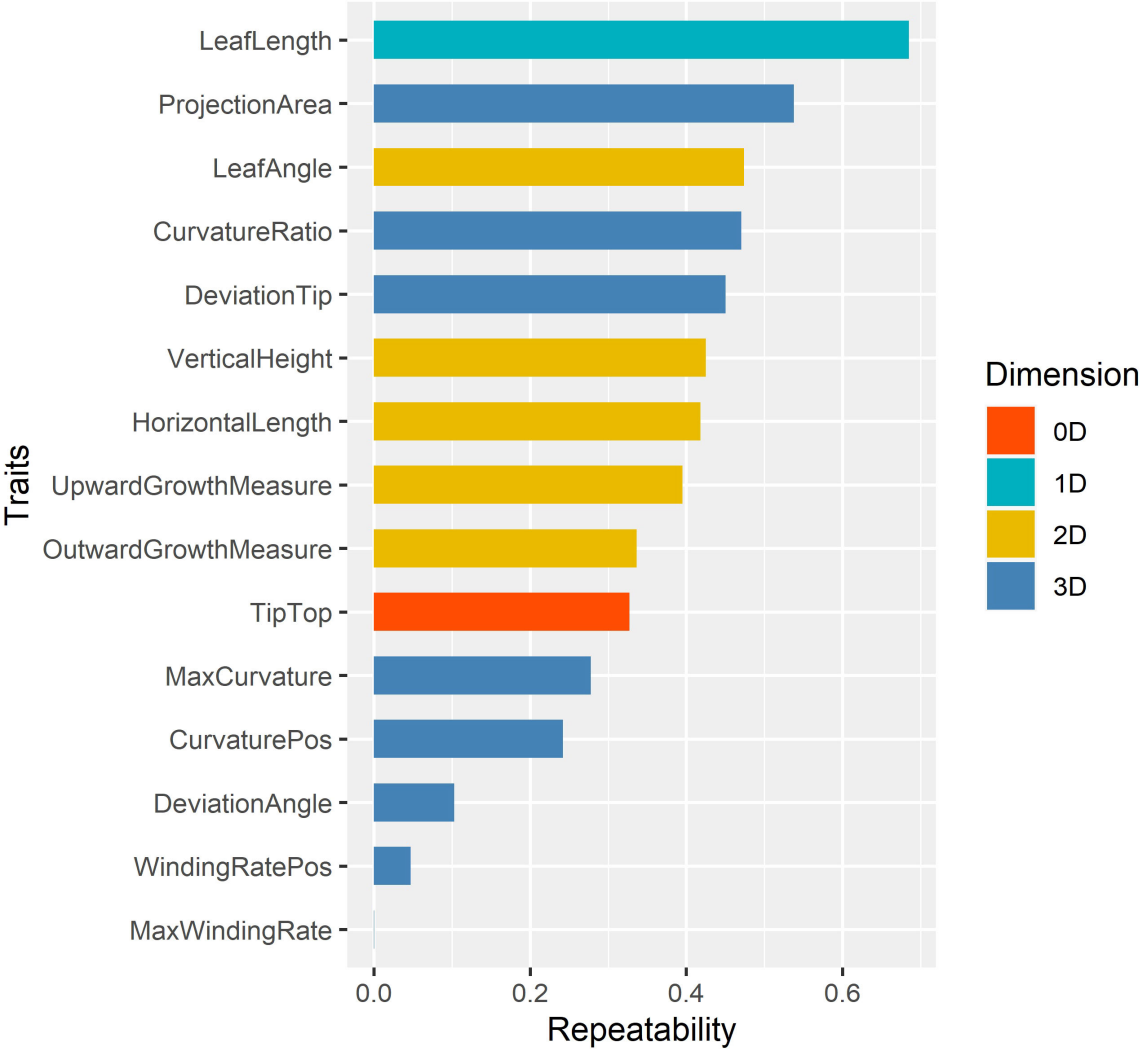
The repeatability of 15 leaf midrib curve traits. The traits are color-coded according to their dimensions.

Repeatabilities of 15 leaf midrib curve traits were calculated to select the key phenotypes (Chen and Lubberstedt, 2010), and the results were shown in **Figure 8**. The analysis result showed that different dimensional traits show different genetic patterns with a wide range of repeatability, from 0.0002 to 0.6855. Among them, the repeatbility of 0D trait TipTop was 0.3273, and the repeatbility of 1D trait LeafLength was the highest (0.6855). The repeatbility of five 2D traits ranged from 0.3365 to 0.4738, and LeafAngle had the highest repeatbility. The repeatbility of eight 3D traits ranged from 0.0002 to 0.5378, and ProjectionArea had the highest repeatbility.

In summary, in order to further elucidate the genetic mechanism of leaf midrib curve-related phenotypic traits, 13 traits with repeatability greater than 0.1 were further investigated by GWAS, except for two traits with extremely low repeatability (WindingRatePos, MaxWindingRate).

### 3.3 Clustering and grouping of phenotypic traits

**Figure 9:**
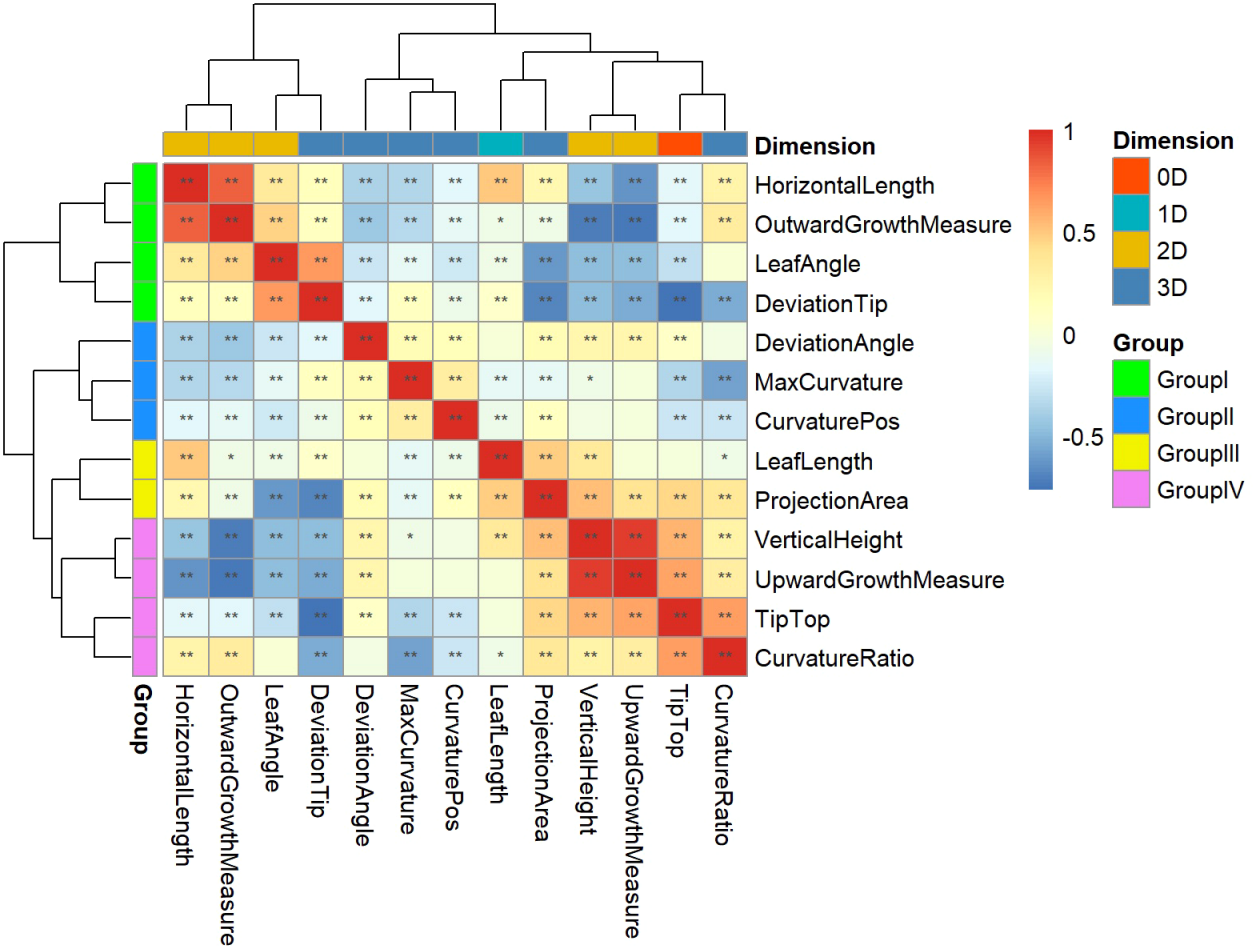
Clustering and grouping of 13 key leaf midrib curve traits. 13 traits covering four dimensions were divided into four groups and marked with different colors: Group I, Group II, Group III, and Group IV. *, P-value<0.05; **, P-value<0.01.

Pearson correlation analysis was performed on 13 key leaf midrib curve traits, and clustering was performed based on the Pearson correlation coefficient, as shown in **Figure 9**. Cluster analysis results showed that 13 traits covering four dimensions could be divided into four groups. Group I contains three 2D traits (HorizontalLength, OutwardGrowthMeasure and LeafAngle) and a 3D trait (DeviationTip). Group II contains three 3D traits (DeviationAngle, MaxCurvature and CurvaturePos). Group III contains a 1D trait (LeafLength) and a 3D trait (ProjectionArea). Group IV contains a 0D trait (TipTop), two 2D traits (VerticalHeight and UpwardGrowthMeasure), and a 3D trait (CurvatureRatio). The above four groups of traits provide a comprehensive characterization of the geometric shape and spatial posture of maize leaf midribs.

The 13 traits from these four groups were analyzed for differences between subpopulations in turn, and it was found that the phenotypic traits differed between subpopulations (**Figure S1**). In Group I, LeafAngle and HorizontalLength (2D traits) were significantly different between TST and SS and between NSS and SS. DeviationTip (3D trait) was significantly different between TST and NSS. While the 2D trait OutwardGrowthMeasure showed no differences between subpopulations. In Group II, MaxCurvature and CurvaturePos (3D traits) were significantly different between TST and SS and between NSS and SS. DeviationAngle (3D trait) was significantly different between TST and NSS. In Group III, LeafLength (1D trait) was significantly different between TST and NSS. ProjectionArea (3D trait) was significantly different between TST and SS and between NSS and SS. In Group IV, Tiptop (0D trait), VerticalHeight (2D trait) and CurvatureRatio (3D trait) were significantly different between TST and NSS. While UpwardGrowthMeasure (2D trait) showed no differences between subpopulations.

### 3.4 Significant loci and candidate genes associated with the leaf midrib curve traits

In this study, an R package “mrMLM” (version 4.0) was used for GWAS to identify SNPs significantly associated with 13 leaf midrib curve traits. Finally, 828 SNPs significantly related to 13 key traits were identified (*P*-value<6.4*e*-07) (**Table 2**). Based on the physical locations of significant SNPs on the B73 reference genome sequence (B73 RefGen_v4), 1365 candidate genes were identified. Among these 828 SNPs, 137 were the most significant (Top1) SNP obtained by each GWAS method or SNP verified by two or more methods, which were annotated to 238 candidate genes (**Table 2**). These highly significant or multi-method verification results were considered to be of high reliability.

**Table 2:** Summary of significant SNPs and candidate genes from GWAS (*Top1, the most significant SNP obtained by each GWAS method.).

Dimension of trait	Meaning	Trait	No. of unique SNPs	No. of unique annotated genes	No. of genes only related to one trait	No. of significant SNPs listed Top 1^*^ or validated by multiple methods	No. of unique annotated genes listed Top1 or validated by multiple methods	No. of genes only related to one trait listed Top 1 or validated by multiple methods
0D	Blade droop or not	TipTop	98	177	140	8	14	12
1D	Biomass	LeafLength	83	143	125	15	27	27
2D	Growth degree	HorizontalLength	62	99	91	17	32	32
2D	Growth degree	OutwardGrowthMeasure	36	59	43	9	14	13
2D	Growth degree	UpwardGrowthMeasure	26	45	29	3	4	1
2D	Growth degree	VerticalHeight	91	168	142	13	22	19
2D	Compactness	LeafAngle	59	106	86	14	22	17
3D	Curvature	CurvaturePos	64	88	78	6	8	8
3D	Curvature	CurvatureRatio	40	72	55	8	16	15
3D	Curvature	DeviationTip	38	67	42	14	26	20
3D	Curvature	MaxCurvature	36	65	55	11	22	21
3D	Deflection	DeviationAngle	144	261	244	10	18	18
3D	Space occupancy	ProjectionArea	82	148	122	13	24	24
		**Summary**	**828**	**1365**	**1252**	**137**	**238**	**227**

**Figure 10:**
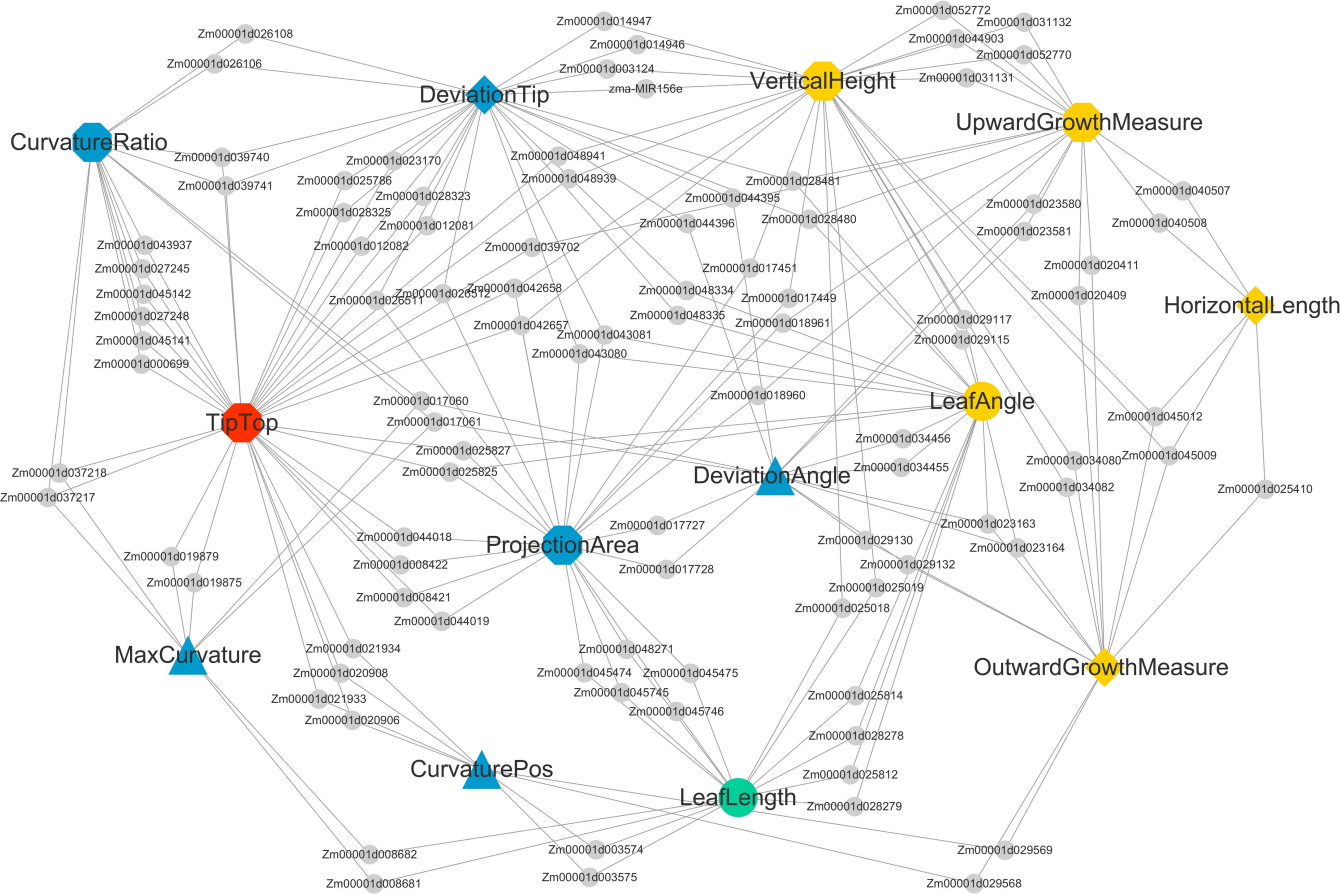
Shared candidate genes among 13 traits of maize ear leaf midrib curves. Traits and genes are shown in different shapes and sizes. Of the 13 large nodes, traits of different dimensions are represented by different colors and shapes. Candidate genes are represented by the small gray circular nodes.

The NCBI Gene database was used to obtain a clearer functional description of these 238 candidate genes. As a consequence, 155 candidate genes were obtained with detailed functional descriptions (**Table S2**). Among these, 29 candidate genes with the highest significant and multi-method validation were regarded as the key findings in this study (**Table 3**). It can be seen that some leaf midrib curve traits had shared SNPs and genes. For example, *GRMZM2G017257* located on chromosome 9 was shared for UpwardGrowthMeasure and VerticalHeight, and *GRMZM5G846140* located on chromosome 3 was shared for LeafAngle and DeviationTip. In addition, based on the 238 candidate gene results it can be seen that there were shared candidate genes between most of the 13 leaf midrib traits and formed a trait-candidate gene network (**Figure 10**). These traits with shared genes or SNPs above are all independent and non-derived, but some of them were significantly positive or negative correlations (*P*-value<0.05). Therefore, it can be speculated that shared genetic loci also exist between phenotype-related traits.

**Table 3:** Detailed functional descriptions of 29 genes annotated by both Top1 and multi-method validated SNPs.

Gene_Symbol	Description	Chromosome	RS	Category	Trait	Trait dimension
GRMZM2G104204	Homeobox-leucine zipper protein ATHB-40	7	chr7.S_166163257	intergenic	TipTop	0D
Zm00001d023435	calmodulin-binding transcription activator 1	10	chr10.S_5458633	intergenic	TipTop	0D
GRMZM5G876003	Ribosomal RNA-processing protein 8-like	4	chr4.S_237386375	intergenic	LeafLength	1D
GRMZM2G336875	40S ribosomal protein S8	4	chr4.S_237386375	intergenic	LeafLength	1D
GRMZM2G095147	glycine-rich protein	5	chr5.S_187085022	intergenic	LeafLength	1D
GRMZM2G129189	endochitinase PR4	5	chr5.S_187085022	intergenic	LeafLength	1D
GRMZM2G074585	Expansin-A11	10	chr10.S_99957462	intergenic	LeafLength	1D
GRMZM2G157313	subtilisin-like protease SBT2.4	10	chr10.S_99957462	intergenic	LeafLength	1D
GRMZM2G170336	40S ribosomal protein S20-2	1	chr1.S_34653763	intronic	LeafAngle	2D
GRMZM5G846140	KH domain-containing protein	3	chr3.S_188669938	intergenic	LeafAngle,DeviationTip	2D, 3D
GRMZM5G890241	LAP4 protein	1	chr1.S_25883345	intergenic	HorizontalLength	2D
Zm00001d028193	AT-rich interactive domain-containing protein 2	1	chr1.S_25883345	intergenic	HorizontalLength	2D
GRMZM2G046583	Nicotinate phosphoribosyltransferase-like protein	10	chr10.S_117902782	intergenic	HorizontalLength	2D
GRMZM2G043737	40S ribosomal protein S10-like	10	chr10.S_117902782	intergenic	HorizontalLength	2D
GRMZM2G072448	Senescence-specific cysteine protease SAG39-like	1	chr1.S_76732537	intergenic	OutwardGrowthMeasure	2D
GRMZM2G075584	P-loop containing nucleoside triphosphate hydrolase superfamily protein	6	chr6.S_87284231	intergenic	OutwardGrowthMeasure	2D
GRMZM2G017257	Kinesin-like protein KCA2	9	chr9.S_6779616	intronic	UpwardGrowthMeasure, VerticalHeight	2D
GRMZM2G157536	lipid binding protein	6	chr6.S_159378178	upstream	CurvaturePos	3D
GRMZM2G174782	SGNH hydrolase-type esterase superfamily protein	4	chr4.S_189178603	intergenic	CurvatureRatio	3D
GRMZM2G109966	protein ABERRANT PANICLE ORGANIZATION 1	9	chr9.S_112493053	intergenic	CurvatureRatio	3D
GRMZM6G901724	metallopeptidase	9	chr9.S_112493053	intergenic	CurvatureRatio	3D
GRMZM2G057084	Serine/threonine-protein kinase GRIK1	10	chr10.S_134400930	intergenic	CurvatureRatio	3D
GRMZM2G014653	NAC domain-containing protein 48	3	chr3.S_173477648	intergenic	DeviationAngle	3D
GRMZM2G049194	MYB-related protein	5	chr5.S_217536124	intronic	DeviationAngle	3D
GRMZM2G035134	Phosphoethanolamine/phosphocholine phosphatase	3	chr3.S_227082662	intergenic	DeviationTip	3D
GRMZM2G120619	chlorophyll a-b binding protein of LHCII type 1	3	chr3.S_227082662	intergenic	DeviationTip	3D
GRMZM2G349243	L-type lectin-domain containing receptor kinase SIT2	3	chr3.S_210051193	intergenic	MaxCurvature	3D
GRMZM2G169654	DNA-binding protein RAV1	3	chr3.S_210051193	intergenic	MaxCurvature	3D
GRMZM5G829738	Polyadenylate-binding protein-interacting protein 3	5	chr5.S_164882040	intergenic	ProjectionArea	3D

### 3.5 Pathways enriched by functional enrichment analysis

**Figure 11:**
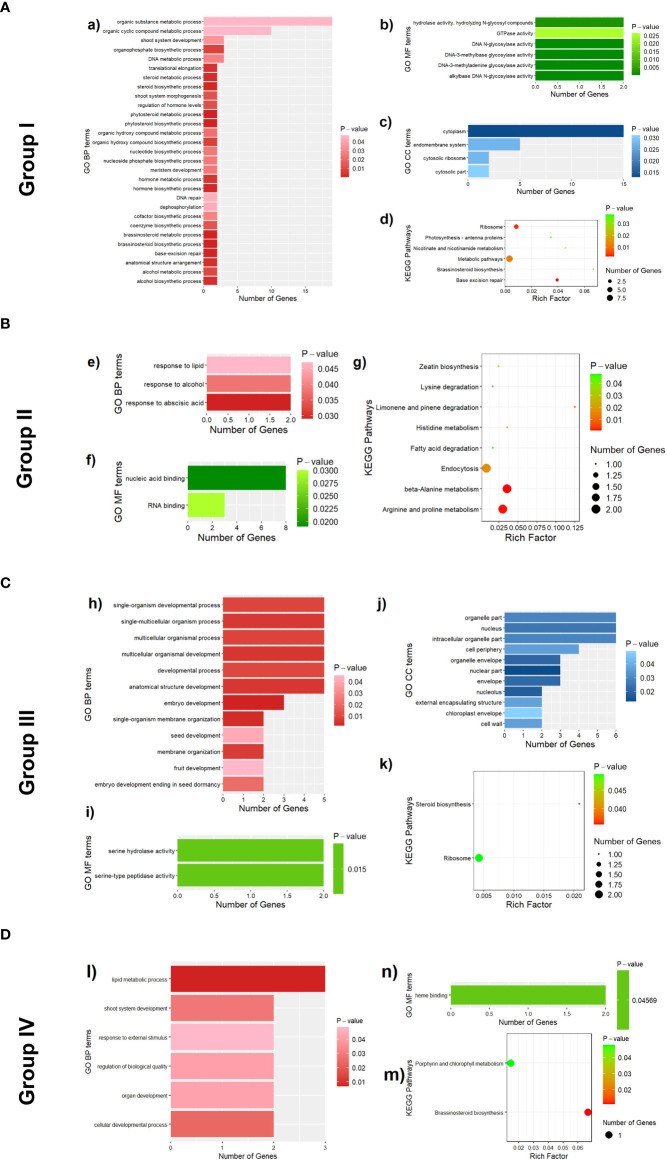
Functional enrichment results of 161 candidate genes associated with 13 leaf midrib curve traits. **(A)** GO terms (a, b and c) and KEGG pathways (d) enriched by candidate genes of traits in Group I. **(B)** GO terms (e and f) and KEGG pathways (g) enriched by candidate genes of traits in Group II. **(C)** GO terms (h, I and j) and KEGG pathways (k) enriched by candidate genes of traits in Group III. **(D)** GO terms (l and n) and KEGG pathways (m) enriched by candidate genes of traits in Group I.

To learn more about the function of candidate genes, a total of 161 candidate genes with detailed functional descriptions for leaf midrib curve traits were enriched according to the three clustering groups separately. For Group I, a total of 39 GO terms and 6 KEGG pathways (*P*-value<0.05) were obtained by enrichment of candidate genes for 4 traits (**Figure 11A**), among which 29 GO terms belonged to GO BP (biological process). In GO BP terms, 8 of the 10 most significant terms were related to plant steroid biosynthesis or metabolism. For example, “phytosteroid biosynthetic process” (GO:0016129, *P*-value=8.2*e*-5), “brassinosteroid biosynthetic process” (GO:0016132, *P*-value=8.2*e*-5), “phytosteroid metabolic process” (GO:0016128, *P*-value=0.0002), “brassinosteroid metabolic process” (GO:0016131, *P*-value=0.0002), “steroid biosynthetic process” (GO:0006694, P-value=0.0011), “hormone biosynthetic process” (GO:0042446, *P*-value=0.0012), “steroid metabolic process” (GO:0008202, *P*-value=0.0022), “alcohol biosynthetic process” (GO:0046165, *P*-value=0.0025). In addition, two pathways related to the anatomical structure were obtained in the GO analysis. One was “anatomical structure arrangement” (GO:0048532, *P*-value=0.0028), which was the process that gave rise to the configuration of the constituent parts of an anatomical structure, and pertained to the physical shaping of a rudimentary structure. The other was “shoot system morphogenesis” (GO:0010016, *P*-value=0.0124), which was a process in which the anatomical structures of the shoot are generated and organized, suggesting that candidate genes of traits in Group I were involved in the generation and organization of the shoot. The most significant pathway in the KEGG analysis was “base excision repair” (zma03410, *P*-value=0.0021), which was consistent with a GO analysis result of “base-excision repair” (GO:0006284, *P*-value=0.0006). In addition, another KEGG pathway “brassinosteroid biosynthesis” (zma00905, *P*-value=0.02029) was also consistent with the GO analysis results.

For Group II, a total of 5 GO terms and 8 KEGG pathways (*P*-value<0.05) were obtained by enrichment of candidate genes for 3 traits (**Figure 11B**), among which 3 GO terms belonged to GO BP. The three GO BP terms were “response to abscisic acid” (GO:0009737, *P*-value=0.029), “response to alcohol” (GO:0097305, *P*-value=0.038), and “response to lipid” (GO:0033993, *P*-value=0.047). It was suggested that most of the candidate genes for the second group of traits were involved in processes in response to abscisic acid, alcohol and lipids, among other substances. The results of the KEGG analysis mainly covered the metabolism of amino acids, such as”beta-Alanine metabolism” (zma00410, *P*-value=0.0011), “Arginine and proline metabolism” (zma00330, *P*-value=0.0014), “Histidine metabolism” (zma00340, *P*-value=0.0232), “Lysine degradation” (zma00310, *P*-value=0.0474), and the synthesis and degradation of other organic substances, such as “Limonene and pinene degradation” (zma00903, *P*-value=0.0073), “Zeatin biosynthesis” (zma00908, *P*-value=0.0334), “Fatty acid degradation” (zma00071, *P*-value=0.0474).

For Group III, a total of 25 GO terms (*P*-value<0.05) were obtained by enrichment of candidate genes for 2 traits (**Figure 11C**), among which 12 GO terms belonged to GO BP. In GO BP terms, several pathways related to developmental process were enriched by genes *GRMZM2G017654*, *GRMZM2G018403*, *GRMZM2G129189*, *GRMZM2G157313* and *GRMZM2G412888*: “Embryo development” (GO:0009790, *P*-value=0.0017), “Multicellular organismal development” (GO:0007275, *P*-value=0.0033), “Anatomical structure development” (GO:0048856, *P*-value=0.0056), “Single-organism developmental process” (GO:0044767, *P*-value=0.0091) and “Developmental process” (GO:0032502, *P*-value=0.0099). In addition, candidate genes (GRMZM2G018403 and GRMZM2G129189) for LeafLength were also enriched in multiple pathways related to development. For example, “Embryo development ending in seed dormancy” (GO:0009793, *P*-value=0.0208), “Seed development” (GO:0048316, *P*-value=0.0411) and “Fruit development” (GO:0010154, *P*-value=0.0453).

For Group IV, 7 GO terms (*P*-value<0.05) were obtained by enrichment of candidate genes for 4 traits (**Figure 11D**), among which 6 GO terms belonged to GO BP. In GO BP terms, the pathway with the highest significance was “Lipid metabolic process (GO:0006629, *P*-value=0.0069). Besides, the other GO BP terms were mainly related to developmental process, such as “cellular developmental process” (GO:0048869, *P*-value=0.0241), “shoot system development” (GO:0048367, *P*-value=0.0286) and “organ development” (GO:0048513, *P*-value=0.0421).

## 4 Discussion

Based on 3D digitizer technology, the key point data of the ear leaf midrib curve of 495 lines of maize association population are collected. The CISCB-spline is constructed, and the smoothed curve has a high degree of coincidence with the natural shape, is naturally smooth, and satisfies the segmental second-order continuity. The LMPS is developed, and 15 phenotypic indexes are extracted from leaf midrib curve data. According to the morphological structure of the leaf midrib curve, these phenotypic indexes are classified into multidimensional phenotypes such as 0-D, 1-D, 2-D and 3-D, and the related phenotypic characteristics such as the relationship between plants and leaves spatial occupancy and spatial growth posture are quantitatively calculated. Based on this method, compared with the original data, the RMSE of leaf length, leaf vertical height and leaf angle decreased by 56%, 60% and 56%, respectively. The results show that the human error of three-dimensional digital data is well corrected.

Obtaining accurate phenotypic data is a critical aspect of phenotypic analysis. The data collected by 3D digital equipment is structured and ordered, which reduces the complexity of phenotype analysis and improves accuracy and efficiency. However, it has its own shortcomings, which are greatly affected by manual operation, especially when carrying out mass sample data collection or obtaining large crop plants, the human error is amplified. The low efficiency of data acquisition is another defect of digitizer technology. At present, some researchers have carried out automatic acquisition of plant point cloud data based on point cloud technology, but to extract high-precision leaf midrib phenotype traits from the cluttered point cloud data, related algorithms or systems cannot be automatically executed, and their robustness is not good either (Wu et al., 2020b). In view of this, the next step is to closely combine the point cloud technology and deep learning technology, and develop an automatic data acquisition system and phenotype extraction system to obtain the refined and high-dimensional phenotype of leaf midribs. In addition, the similarity of leaf midribs between different varieties and more subtle phenotypic characteristics such as segmented leaf midribs also need to be deeply excavated and studied.

By using 3D digitizer equipment, it is possible to obtain accurate three-dimensional data on the ear leaf midrib curves of the maize association population, while different dimensional traits can be obtained. However, the interpretability of traits still needs to be explored in depth. In recent years, GWAS has become an effective method to systematically identify the genetic components of complex traits of maize leaf. There are numerous studies (Guo et al., 2015; Li et al., 2015; Lu et al., 2018; Wang et al., 2018; Gao et al., 2021; Zhang and Huang, 2021) with QTL or GWAS results for a number of target traits related to leaves, suggesting that this technology is effective in mining candidate genes that control these traits in maize. For example, leaf angle is an important determinant of plant architecture, a F3:4 recombinant inbred lines (RIL) population derived from a cross between B73 and Zheng58 were used to map QTL for leaf angle, and a sum of eight QTLs were detected on chromosome 1, 2, 3, 4 and 8 (Zhang and Huang, 2021). To extend the understanding of the genetic mechanisms involved in leaf-related traits, three RIL populations including 538 RILs were genotyped by genotyping-by-sequencing (GBS) method, and a total of 45 QTLs were detected for four leaf architecture traits by using joint linkage mapping across the three populations (Li et al., 2015). Besides, leaf orientation value is also an important trait affecting planting density. It has been reported that 33 significantly SNPs (*P*-value<1*e*-6) with leaf angle and leaf orientation value were identified by GWAS (Lu et al., 2018).

In the present study, GWAS analysis was performed on 13 key leaf midrib curve traits with genotypic data, and 155 candidate genes were obtained with detailed functional descriptions (**Table S2**). For TipTop and DeviationTip, two traits that were significantly and negatively correlated (**Figure 9**), *Dwf4* is a common candidate gene for both traits. Previous studies showed that *Dwf4* encoding putative cytochrome P450 superfamily protein was associated with changes in leaf angle in cereal crop species, such as maize (Mantilla-Perez and Salas Fernandez, 2017; Dzievit et al., 2019; Han et al., 2022). TipTop indicated whether the leaf tip is the highest point of the leaf midrib curve, and DeviationTip represents the ratio of drooping length to LeafLength, and the drooping length is the length from the highest point to the leaf tip along the leaf midrib, both of them could have an effect on the leaf angle. Hence, *Dwf4* may participate in the regulation of DeviationTip and TipTop. The previous study (Zhang and Huang, 2021) also confirmed that a candidate gene (*Zm00001d005888*) of leaf angle was enriched in the GO term “response to abscisic acid (GO:0009737)”, which is consistent with the functional enrichment analysis results of Group II in our study. Here, GO:0009737 was enriched by *GRMZM2G014653* and *GRMZM2G083546*. *GRMZM2G014653*, which were candidate genes of DeviationAngle and CurvaturePos, separately. Among these, DeviationAngle suggested the deviation angle between the leaf midrib and the main plane of the midrib, which was closely related to the leaf angle. Accordingly, it is deduced that candidate genes of the leaf midrib curve traits obtained in this study might provide new genetic loci for maize leaf spatial structure to improve the plant type of maize.

In this study, an innovative multidimensional phenotype quantitative description and calculation method for maize leaf midrib curves was proposed. A breakthrough has been achieved in the acquisition of high-throughput phenotypes of leaf venation profiles in large groups of maize ears based on digitizers. This study can provide a wealth of phenotypic information for maize researchers. In addition, a combined phenotype-genotype study was conducted to identify candidate genes related to the traits of the ear leaf midrib curve, providing a theoretical basis for the analysis of the genetic mechanism of the formation of important phenotypes related to the ideal plant type of maize and the breeding of dense and high-yielding varieties.

Project name: LMPS

Project home page: https://github.com/wusheng999000/LMPS.git

Operating systems: Windows

Programming languages: C++

## Data availability statement

The raw data supporting the conclusions of this article will be made available by the authors, without undue reservation. For the availability of supporting source code and requirements, see LMPS, https://github.com/wusheng999000/LMPS.git.

## Author contributions

SW implemented the algorithm for leaf midrib smoothing and phenotypic analysis, and the system development work. JW implemented the analysis of phenotypic data and GWAS procedure. SW and JW drafted and revised the manuscript. YaZ designed and responsible for the experimental planning and planting, and participated in the design and revision of the manuscript. WW, YiZ, XL and CW participated in the design of the manuscript. WW, LK and CB were involved in data acquisition. XG and CZ conceived and coordinated the design of the study. SW, JW and YaZ contributed equally to this work. All authors contributed to the article and approved the submitted version.

## Funding

This research was funded by the Construction of Collaborative Innovation Center of Beijing Academy of Agricultural and Forestry Sciences (KJCX201917), the National Natural Science Foundation of China (31871519 and 32071891), Science and Technology Innovation Special Construction Funded Program of Beijing Academy of Agriculture and Forestry Sciences (KJCX20210413).

## Conflict of interest

The authors declare that the research was conducted in the absence of any commercial or financial relationships that could be construed as a potential conflict of interest.

## Publisher’s note

All claims expressed in this article are solely those of the authors and do not necessarily represent those of their affiliated organizations, or those of the publisher, the editors and the reviewers. Any product that may be evaluated in this article, or claim that may be made by its manufacturer, is not guaranteed or endorsed by the publisher.

## Supplementary material

Supplementary Table 1 The leaf midrib phenotypic indexes and their calculation description.

Supplementary Table 2 155 candidate genes of 13 key leaf midrib curve traits with detailed functional descriptions.

Supplementary Figure 1 The trait variation between subpopulations (TST, NSS, SS and mixed) for the 15 phenotypic traits (divided into four groups).

Algorithm file S1 The calculation formula of leaf midrib curve winding rate.

Algorithm file S2 Algorithm flow of the Leaf midrib phenotype system (LMPS).

Algorithm file S3 The leaf midrib classification algorithm.
