# Supplementary figures and images for: Characterization and genetic dissection of maize ear leaf midrib acquired by 3D digital technology
Source: Front Plant Sci. 2022 Dec 1;13:1063056. doi: 10.3389/fpls.2022.1063056 (PMC9754214; doi:10.3389/fpls.2022.1063056)

GroupI

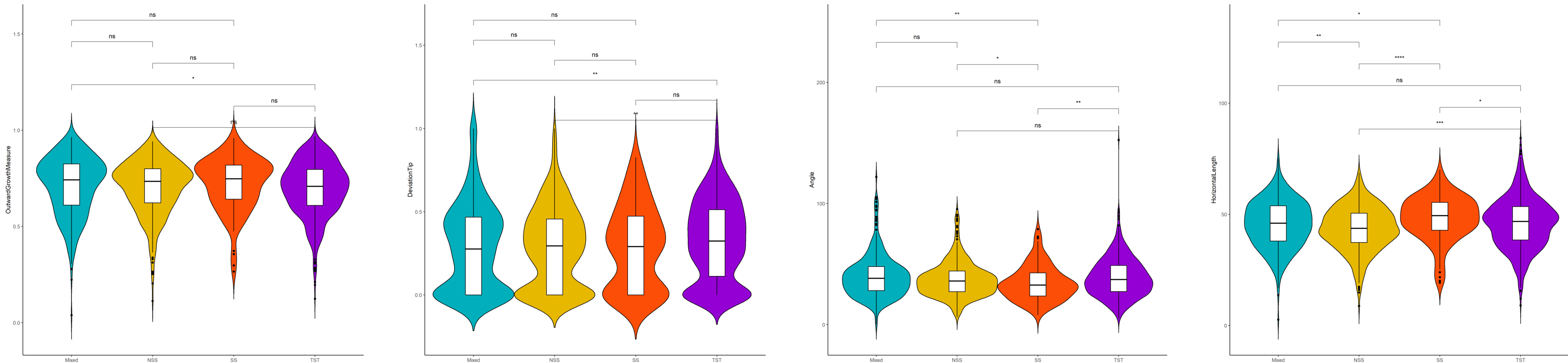

GroupII

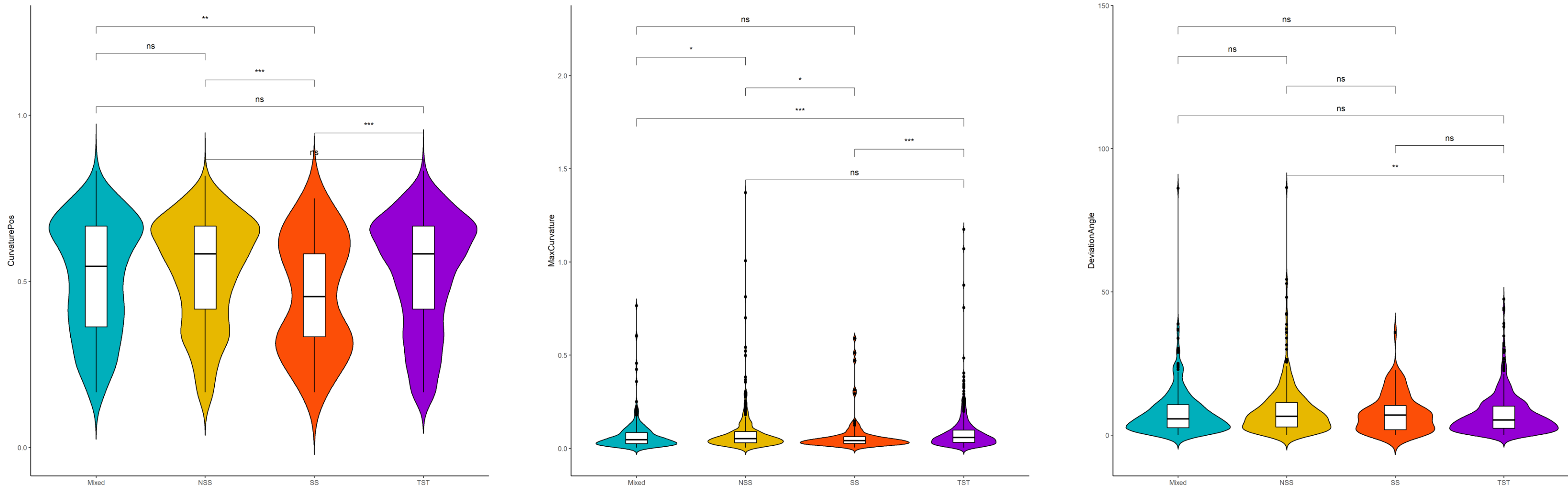

GroupIII

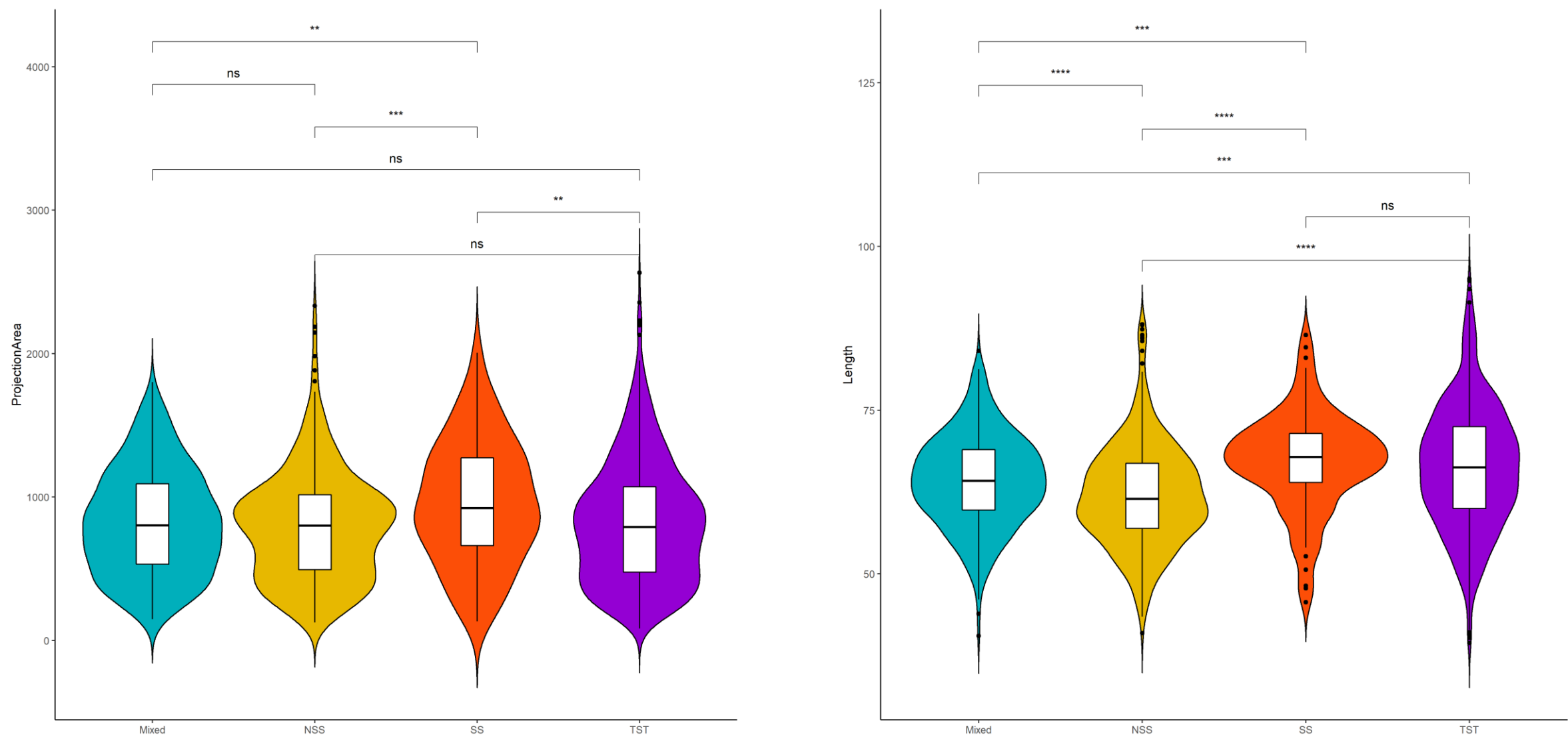

GroupIV

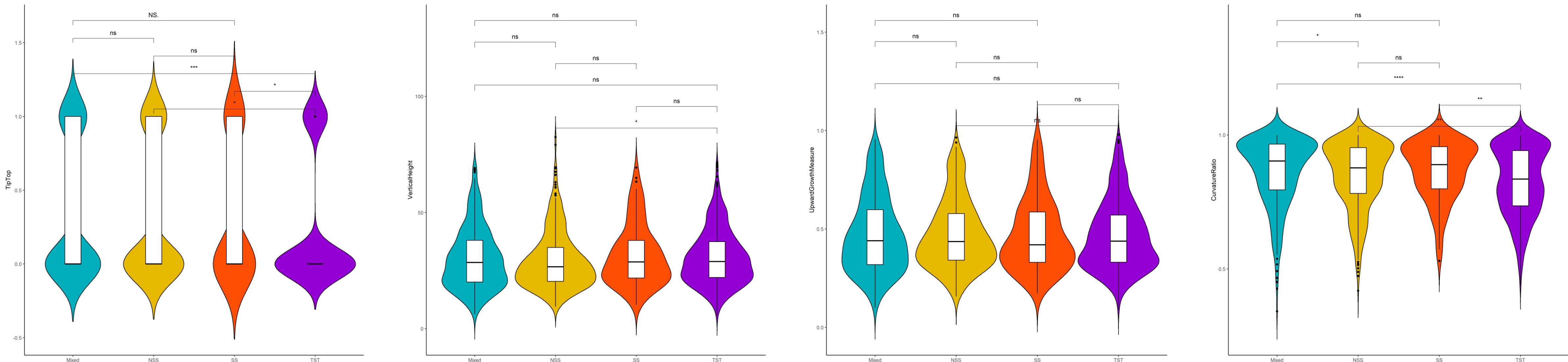

Supplement: Supplementary Table 1 — The leaf midrib phenotypic indexes and their calculation description. [file DataSheet_1.zip › Data Sheet 1.PDF]
