# Supplementary material for: Characterization and genetic dissection of maize ear leaf midrib acquired by 3D digital technology
Source: Front Plant Sci. 2022 Dec 1;13:1063056. doi: 10.3389/fpls.2022.1063056 (PMC9754214; doi:10.3389/fpls.2022.1063056)
Supplement: Supplementary Table 1 — The leaf midrib phenotypic indexes and their calculation description. [file DataSheet_1.zip › Table 1.DOC]

| **Table S1: leaf midrib phenotypic indexes and their calculation description** | | | | |
| --- | --- | --- | --- | --- |
| Phenotypic Index | Calculation Description | Unit | Dimension | Schematic |
| TipTop | Whether the leaf tip is the highest point of the leaf midrib curve | bool | 0-D | / |
| LeafLength | The sum of the lengths of the segmented line segments from the start point of the leaf midrib curve to the leaf tip point | cm | 1-D | Figure 4a |
| LeafAngle | Angle between base segment of the leaf midrib curve and stem | o | 2D | Figure  4b |
| VerticalHeight | The vertical distance between the highest point and the lowest point of the leaf midrib curve | cm | 2D | Figure  4c |
| UpwardGrowthMeasure | *VerticalHeight* / *Length* | / | 2D | / |
| HorizontalLength | Projection length of leaf midrib curve in x-axis direction | cm | 2D | Figure  4d |
| OutwardGrowthMeasure | *HorizontalLength* / *Length* | / | 2D | / |
| DeviationTip | The ratio of drooping length to *LeafLength*, and the drooping length is the length from the highest point to the leaf tip along the leaf midrib. | / | 3D | Figure 4e |
| CurvatureRatio | The ratio of natural length to *LeafLength*, and the natural length is the linear distance from leaf tip to leaf base. | / | 3D | Figure  4f |
| MaxCurvature | The maximum curvature value in the key point of the leaf midrib curve. | / | 3D | / |
| CurvaturePos | The ratio of curvature length to *LeafLength*, and the curvature length is the length from the leaf tip to the maximum curvature key point along the leaf midrib. | / | 3D | / |
| DeviationAngle | The deviation angle between the leaf midrib and the main plane of the midrib. | o | 3D | Figure 4g |
| MaxWindingRate | The maximum Winding Rate value in the key point of the leaf midrib curve. See Algorithm file S1 for the calculation formula of winding rate of key nodes of leaf midrib curve. | / | 3D | / |
| MaxWindingRatePos | The ratio of winding Rate length to *LeafLength*, and the winding rate length is the length from the leaf tip to the maximum winding Rate key point along the leaf midrib. | / | 3D | / |
| ProjectionArea | Sum of segmented trapezoidal areas formed by leaf midrib curve node and its projection node in *XOY* plane. | cm^2^ | 3D | Figure 4h |

**Algorithm file S1: the calculation formula of leaf vein curve winding rate**

The growth of maize leaves is affected by gravity and phototaxis. Therefore, in three-dimensional space, leaf vein curves not only have curved spatial attitude characteristics, but also distorted attitude characteristics. The twist degree is one of the varietal characteristics of the leaf veins of maize leaves.Curve winding rate is a general method for quantitative calculation of three-dimensional curve distortion. Based on the calculation formula of cubic spline curvature, the calculation formula of leaf vein winding rate is introduced as follows:

（8）

Combined with the parameter equation 5 of the curve, the parameterized calculation formula of the winding rate of the leaf vein curve is obtained as follows:

$\tau(t)=\frac{x'''(t)(z''(t)y'(t)-(y''(t)z'(t))+y'''(t)(x''(t)z'(t)-(z''(t)x'(t))+z'''(t)(y''(t)x'(t)-x''(t)y'(t))}{(z''(t)y'(t)-(y''(t)z'(t))^{2}+(x''(t)z'(t)-(z''(t)x'(t))^{2}+(y''(t)x'(t)-x''(t)y'(t))^{2}}$ （9）

Further, the winding rate value of each interpolation node of the leaf vein curve under the cubic B-spline interpolation curve is calculated by formula 7.

It should be noted that in order to avoid the localization of curve curvature and winding rate and improve their global representation accuracy, the smoothed leaf vein nodes are sparsely sampled, so that the number of nodes brought into the calculation is controlled within 20.

**Algorithm file S2: Algorithm flow of the Leaf midrib phenotype system (LMPS)**

**Algorithm file S3: The leaf midrib classification algorithm**

**Figure S1**

The trait variation between subpopulations (TST, NSS, SS and mixed) for the 15 phenotypic traits (divided into three groups).
